# *Lactobacillus plantarum* Alleviates Obesity by Altering the Composition of the Gut Microbiota in High-Fat Diet-Fed Mice

**DOI:** 10.3389/fnut.2022.947367

**Published:** 2022-06-30

**Authors:** Yong Ma, Yanquan Fei, Xuebing Han, Gang Liu, Jun Fang

**Affiliations:** Hunan Provincial Engineering Research Center of Applied Microbial Resources Development for Livestock and Poultry, College of Bioscience and Biotechnology, Hunan Agricultural University, Changsha, China

**Keywords:** high fat diet, *Lactobacillus plantarum*, SCFA, fat metabolism, intestinal microbes

## Abstract

Metabolic disorders and intestinal flora imbalance usually accompany obesity. Due to its diverse biological activities, *Lactobacillus plantarum* is widely used to alleviate various diseases as a probiotic. Here, we show that *L. plantarum* can reduce the body weight of mice fed high-fat diets, reduce fat accumulation, and enhance mice glucose tolerance. Our results show that *L. plantarum* can significantly reduce the expression of DGAT1 and DGAT2, increase the expression of Cpt1a, and promote the process of lipid metabolism. Further data show that *L. plantarum* can increase the SCFA content in the colon and reverse the intestinal flora disorder caused by HFD, increase the abundance of *Bacteroides*, and *Bifidobacteriales*, and reduce the abundance of *Firmicutes* and *Clostridiales*. Finally, through Pearson correlation analysis, we found that *Bacteroides* and SCFAs are positively correlated, while *Clostridiales* are negatively correlated with SCFAs. Therefore, we believe that *L. plantarum* can regulate the structure of the intestinal microbial community, increase the production of SCFAs and thus regulate lipid metabolism.

## Introduction

Accumulating evidence points a high-fat diet will increase the potential risk of metabolic diseases (1). Long-term excessive intake of high-energy foods can lead to the accumulation of fat, which can lead to obesity. Obesity and the development of metabolic disorders have caused great troubles for the world health security authorities. According to the World Health Organization, over the past 30 years, more than 9 million children have been considered overweight in the world (https://www.who.int/zh). According to the U.S. Centers for Disease Control and Prevention, the national obesity rate from 2017 to 2018 was 42.4% (https://www.cdc.gov/obesity/data/adult.html). The obesity caused by this long-term high-fat diet will further cause metabolic diseases such as diabetes and non-alcoholic fatty liver (2, 3). Most importantly, HFD-induced obesity is usually accompanied by changes in the structure of the gut microbial community (4), and gut microbes are a vital part of the host's energy intake (5). This reduction in gut microbial diversity and changes in the abundance of specific microorganisms may be determinants of lipid metabolism and obesity. Intestinal microbes are involved in the process from lipid synthesis and absorption to lipid metabolism in obesity (6). This also causes obesity to be accompanied by changes in the intestinal microbial community. The dominance of *Bacteroides* in normal individuals is caused by the fact that *Bacteroides* can ferment the dietary fiber that the host cannot digest and absorb for energy. However, when fat accumulates, this microorganism community will change, and the abundance of *Firmicutes* will increase significantly in obese hosts (7). Changes in the community structure of intestinal microbes can alter the secretion of intestinal hormones, including peptide YY, GLP-1, leptin, and weaken their effects of inhibiting gastric emptying, reducing appetite, and increasing satiety (8).

With a deep understanding of intestinal microbes, the mechanism of action of probiotics and host flora has been well known by people. Probiotics can produce anti-pathogenic microbial products, which are biologically compatible with the host or interact directly with the host's immune cells, regulate host metabolism, etc. (9). As a probiotic, *Lactobacillus plantarum* has been used to treat and alleviate various diseases due to its diversified functions (10). Through genome-wide association analysis and non-targeted metabolomics analysis, Mao et al. found that *L. plantarum* and its metabolites can significantly increase the level of the neurotransmitter γ-aminobutyric acid in the hippocampus (11). *L. plantarum* has shown an irreplaceable role in intestinal health. *L. plantarum* can activate PPAR-α and restore the structure of mitochondria and ultimately repair the intestinal barrier (12). Further studies have found that bacteriocin, a metabolite of *L. plantarum*, can prevent the loss of Caco-2 cell transcellular permeability and inhibit the expression of inflammatory factors (13). Salomé-Desnoulez et al. developed fluorescent *L. plantarum* strains to track and monitor its dynamic role in intestinal inflammation and found that *L. plantarum* mainly colonizes the intestinal lumen and mucous layer and directly acts on damaged epithelial cells (14). Recent experiments have shown that most of the probiotics acting on the intestine can inhibit obesity, so we suspect that *L. plantarum*, with multiple biological activities, plays an essential role in lipid metabolism.

## Materials and Methods

### Animal and Experimental Design

**Figure 1:**
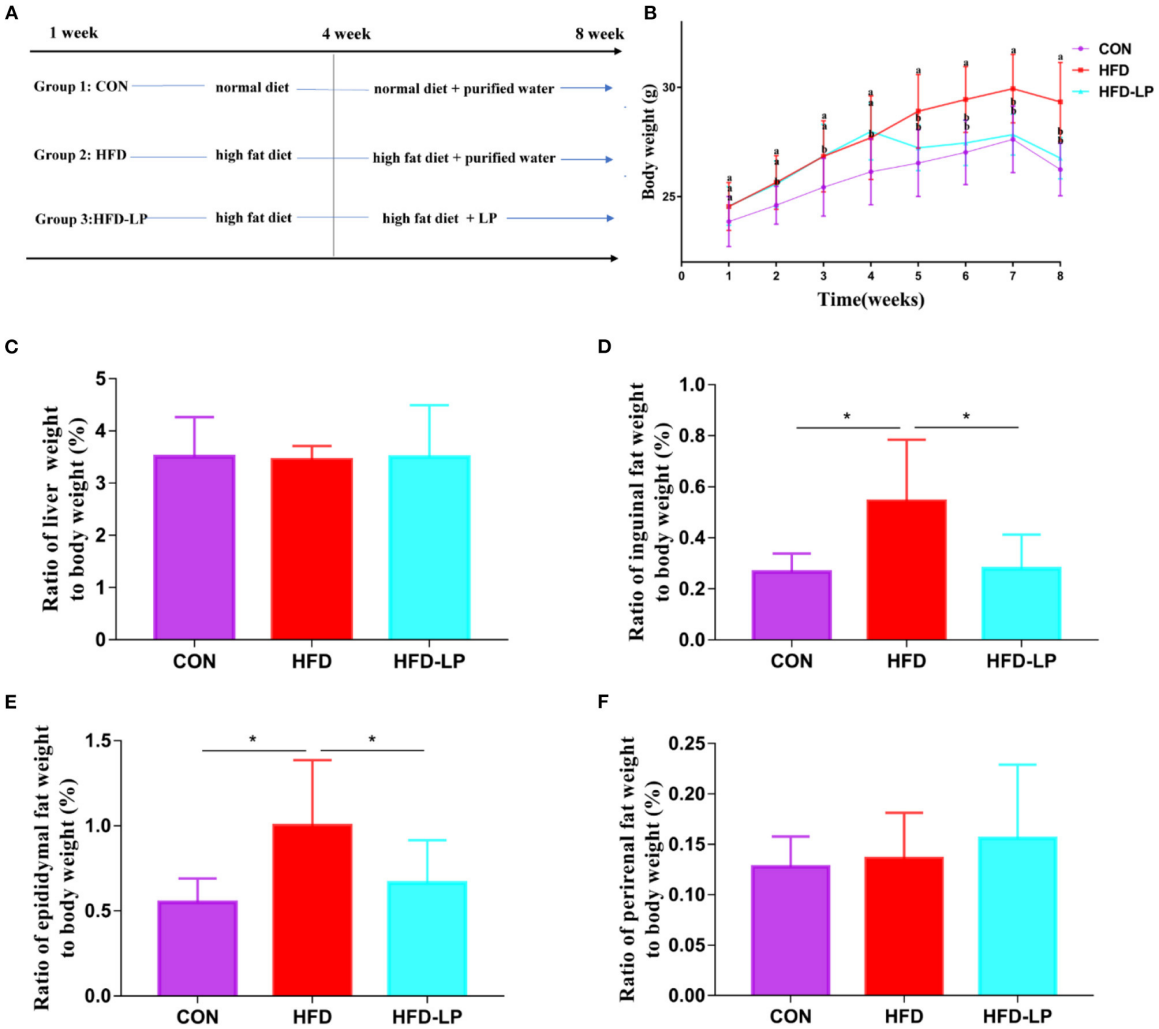
Effect of *Lactobacillus plantarum* on body weight, fat and internal organs of obese mice. **(A)** the experimental process; **(B)** the body weight; **(C)** the ratio of liver weight to body weight; **(D)** the ratio of Inguinal fat weight to body weight; **(E)** the ratio of Epididymis fat weight to body weight; **(F)** the ratio of perirenal fat weight to body weight. Data are mean ± SD (*n* = 8) and analyzed by one-way ANOVA. *p < 0.05.

This animal experiment completely follows the Guidelines for Care and Use of Laboratory Animals of Hunan Agricultural University. Twenty-four 8-week-old C57BL/6J mice [purchased from Hunan Sileike Jingda Co (Changsha, China)] with an average weight of 18 g were acclimated for 7 days in a sterile environment. They were divided into three groups with 8 mice in each group by completely random allocation. The first group was treated with the basal diet (CON), the second group was treated with high-fat diet (HFD), and the third group was treated with *L. plantarum* and high-fat diet (HFD-LP). During the experiment, mice can drink and eat freely. In 1–4 weeks, the HFD group and HFD-LP group were fed with high-fat diets. We administered *L. plantarum* (1 × 10^8^ cfu) to mice in the HFD-LP group for 5–8 weeks, and the other two groups were administered the same dose of normal saline (Figure 1A). Collection of mice epididymal adipose tissue: the mouse testis was found in the lower abdomen and lifted with tweezers. The white fat attached to it was epididymal fat and stripped along the vas deferens to the end of the testis. Inguinal fat: Inguinal fat was cut bluntly between the skin and muscle of the hind limb of mice with ophthalmic scissors, and the skin was capped at the tip of the scissors to avoid cutting off subcutaneous fat. Then the skin was longitudinally cut to expose the inguinal fat, the inguinal lymph nodes were stripped, and the adipose tissue was cut off. Perirenal fat: The kidney was lifted with tweezers and all adipose tissues were cut off along the surface of the renal fascia. All adipose tissues were weighed using a one-tenth balance. Serum samples were collected for serologic analysis. Liver tissue, intestinal contents, and mouse faces were collected and immediately snap-frozen in liquid nitrogen, then transferred and stored in a −80° refrigerator.

### Glucose and Insulin Tolerance Tests

The intraperitoneal glucose tolerance test (IPGTT) was performed after the mice were fasted for 6 h. Each mouse was injected intraperitoneally with glucose (2 g/kg body weight), and the blood glucose of the mice tail vein was measured with a blood glucose meter for the next 0, 15, 30, 60, and 120 min. The insulin tolerance test (ITT) was performed after the mice were fasted for 6 h. Each mouse was injected intraperitoneally with insulin (0.75 U/kg body weight), and the blood glucose of the mice tail vein was measured with a blood glucose meter for the next 0, 15, 30, 60, and 120 min.

### Fat Histopathology

Use gradient concentration of xylene and ethanol solution to dehydrate and transparent adipose tissue. Embed the tissue block with melted paraffin, slice the wax block and stain it with Hematoxylin and Eosin, finally use alcohol for dehydration, and add neutral gum to mount the slide.

### Serologic Analysis

The content of alanine aminotransferase, aspartate aminotransferase, blood glucose, and triglyceride, high-density lipoprotein and low-density lipoprotein (n=8) was measured using kits and a microplate reader (14041717, VT, United States).

### Real-Time Fluorescence-Based Quantitative PCR

Take the liver tissue and grind it with liquid nitrogen, grind it into powder, transfer it into a 1.5 ml EP tube and mix with 1 ml Trizol, and centrifuge at 12,000 g for 10 min at 4°C. Add 120 ul of chloroform to the supernatant, centrifuge for 15 min at 4°C and 1,200 r, take 300 ul of supernatant into another centrifuge tube, add an equal volume of isopropanol to the supernatant, centrifuge at 4°C, 1,000 r for 10 min, remove the supernatant, add 600 ul of 75% ethanol to wash the precipitate, centrifuge at 8,000 r, 4°C for 10 min, remove the ethanol, add an appropriate amount of DNAase Free Water, and use a reverse transcription kit (gDNA Eraser) for reverse transcription. The resulting cDNA was analyzed by conducting the real-time quantitative polymerase chain reaction with a SuperReal PreMix Plus (SYBR Green) reagent kit (TIANGEN, Beijing, China) with various primers (Table 1).

**Table 1:** Primers for this study.

**Primers**	**Nucleotide sequence of primers (5'−3')**
β-actin	F: TGTCCACCTTCCAGCAGATGT
	R: AGCTCAGTAACAGTCCGCCTAGA
DGAT 2	F: AGTGGCAATGCTATCATCATCGT
	R: TCTTCTGGACCCATCGGCCCCAGGA
DGAT 1	F: TTCCGCCTCTGGGCATT
	R: AGAATCGGCCCACAATCCA
Cpt1a	F: CAGTCGACTCACCTTTCCTG
	R: CATCATGGCTTGTCTCAAGTG
TNF-α	F: ATGAGAAGTTCCCAAATGGC
	R: CTCCACTTGGTGGTTTGCTA
IL-6	F: CCTCTCTGCAAGAGACTTCCAT
	R: AGTCTCCTCTCCGGACTTGT
IL-1β	F: TGCCACCTTTTGACAGTGATG
	R: AAGGTCCACGGGAAAGACAC

### SCFA Content in Feces

The analysis was performed using HP7890B gas chromatograph (Agilent) equipped with flame ionization detector and DB-FF capillary column (internal diameter 0.25 mm, length 30 m, film thickness 0.5 μm; temperature 40–250°C; Agilent Technologies, USA). The temperature of the injector and detector are 200 and 270°C, respectively. The carrier gas uses high-purity and high-purity nitrogen with a flow rate of 40 ml/min. Heating program: the initial temperature is 80°C, hold for 0.5 min, 5°C/min rise to 130°C, hold for 2 min, and then rise at 20–240°C/min, hold for 1 min. Confirm the types of short-chain fatty acids and calculate the content of short-chain fatty acids by comparing the retention time and peak area of the sample and the standard. The content of short-chain fatty acids is expressed in μg/g chyme.

### 16S Ribosomal RNA Amplicon Sequencing

Stool samples were extracted from microbial DNA using QIAamp DNA Stool Mini Kit (QIAGEN, Hilden, Germany). The sequence of the extracted DNA V3-V4 region was analyzed by high-throughput sequencing technology. First, the purified DNA was used as a template, and the universal primers 357F 5'-ACTCCTACGGRAGGCAGCAG-3' and 806R 5'-GGACTACHVGGGTWTCTAAT-3' primers were used for PCR amplification, and these primers were also fused with part of Miseq sequencing primers. The amplified products were subjected to 1.2% agarose gel electrophoresis, the samples with good results were run on 2% agarose gel, and the electrophoresis bands were cut and recovered, and the recovered product was used as a template for PCR amplification (8 cycles). Add adapters, sequencing primers, and tag sequences required for sequencing on the Illumina platform at both ends of the target fragment. Use AxyPrepDNA gel recovery kit (AXYGEN company) to recover all PCR products, and use FTC-3000TM Real-Time PCR instrument for fluorescence quantification, complete library construction after equal molar ratio mixing, and computer sequencing (illumina miseq PE300). Analyze the offline data, use mothur (Version 1.33.3) to analyze α diversity and β diversity, and use R language to map the species composition at the level of the phyla, the family, the genera and the species, and the species differences between groups.

### Data Analysis

All data in this study were analyzed using SPSS 22.0, expressed as mean ± standard error of the mean (SEM). The difference between the means of the groups was analyzed using a one-way ANOVA pair and evaluated using Tukey multiple comparisons. *P* < 0.05 was regarded as a significant difference.

## Results

### *Lactobacillus plantarum* Can Reduce HFD-Induced Body Weight and Fat Gain

Starting from the second week, the weight of the mice in the HFD and HFD-LP groups fed with high-fat diets was significantly higher than that in the CON group fed with ordinary diets, and the gap reached the largest in the fourth. From the fifth week, we gave the HFD-LP group an intragastric administration of *L. plantarum*. The bodyweight of the HFD-LP group was significantly lower than that of the HFD group and dropped to the same level as the CON group (Figure 1B). There was no significant difference in the ratio of the liver (Figure 1C) and perirenal fat weight (Figure 1F) to body weight (%) between the three groups. Compared with the HFD group, the ratio of inguinal fat (Figure 1D) and epididymal fat (Figure 1E) weight to body weight (%) of the CON and HFD-LP groups was significantly reduced.

### *Lactobacillus plantarum* Can Enhance Glucose Tolerance, Insulin Resistance and Reduce Adipocyte Size in Obese Mice

**Figure 2:**
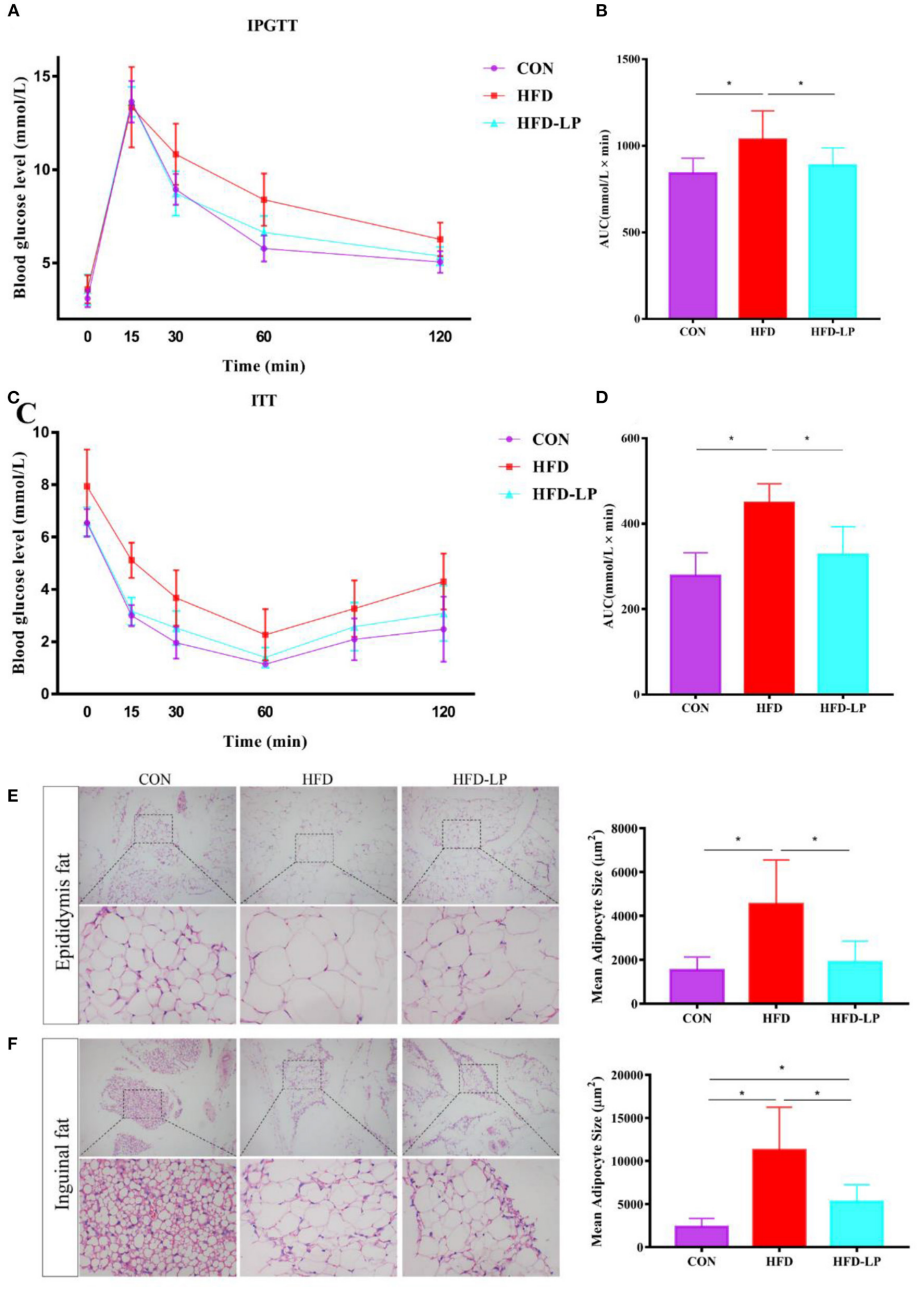
Effect of *Lactobacillus plantarum* on glucose tolerance, insulin resistance and fat morphology in obese mice. **(A)** Plasma glucose level in mouse glucose tolerance test; **(B)** The area under the glucose curve (AUC) using the trapezoidal rule; **(C)** Plasma glucose level in mouse insulin tolerance test; **(D)** The area under the glucose curve (AUC) using the trapezoidal rule. **(E)** H&E staining on epididymal fat depots. Representative sections from inguinal fat depots of all groups (400 ×). Histological analysis of mean adipose cell size. **(F)** H&E staining on inguinal fat depots. Representative sections from inguinal fat depots of all groups (400 ×). Histological analysis of mean adipose cell size. Data are mean ± SD (*n* = 8) and analyzed by one-way ANOVA. *p < 0.05.

In the intraperitoneal glucose tolerance test, we found that all mice had a significant increase in blood glucose after 15 min of intraperitoneal injection of glucose, but within 30–120 min, compared with the HFD group, the mice blood glucose of CON group and the HFD-LP group decreased significantly (Figure 2A). The area under the glucose curve (AUC) of the HFD group mice was significantly larger than that of the CON group and HFD-LP group (Figure 2B). In the insulin tolerance test, we found that compared with the CON group and the CON-LP group, the initial blood glucose of the HFD group mice was significantly increased, and the insulin sensitivity was significantly reduced (Figure 2C). The area under the glucose curve (AUC) of the HFD group mice was significantly larger than that of the CON group and HFD-LP group (Figure 2D).

We performed HE staining sections on epididymal fat and inguinal fat of mice and found that compared with the CON group and the CON-LP group, the size of the adipocytes in the epididymal fat of the HFD group increased significantly (Figure 2E). The size of the adipocytes in the inguinal fat of the CON group was significantly smaller than that of the HFD group and HFD-LP group, while the size of the adipocytes in the inguinal fat of the HFD-LP group was significantly smaller than that of the HFD group (Figure 2F).

### *Lactobacillus plantarum* Can Improve the Biochemical Indexes of Obese Mice

**Figure 3:**
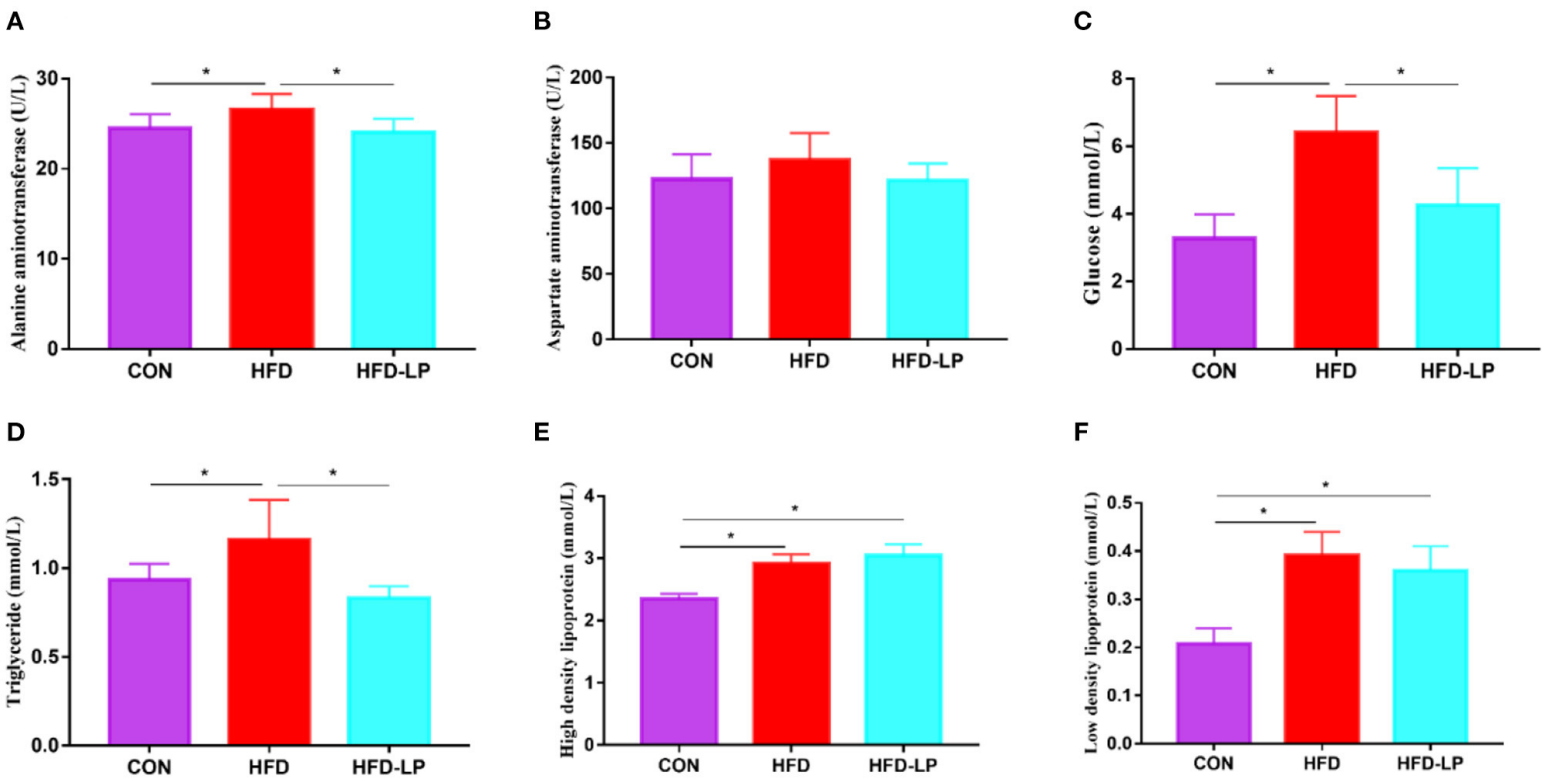
Effects of *Lactobacillus plantarum* on the biochemical indexes of obese mice **(A)** the content of alanine aminotransferase in serum; **(B)** the content of aspartate aminotransferase in serum; **(C)** the Blood glucose level; **(D)** the content of triglyceride in serum; **(E)** the content of high-density lipoprotein in serum; **(F)** the content of low-density lipoprotein. Data are mean ± SD (*n* = 8) and analyzed by one-way ANOVA. *p < 0.05.

The mouse serum biochemical indicators showed that the serum levels of alanine aminotransferase, blood glucose, and triglyceride in the HFD group were significantly higher than those in the CON group and the HFD-LP group (Figures 3A,C,D). There was no significant difference in the content of aspartate aminotransferase in mouse serum among the three groups (Figure 3B). The HFD group and HFD-LP group mice serum high-density lipoprotein and low-density lipoprotein levels were significantly higher than those in the CON group (Figures 3E,F).

### *Lactobacillus plantarum* Can Improve Gene Expression and Substance Content in the Liver

**Figure 4:**
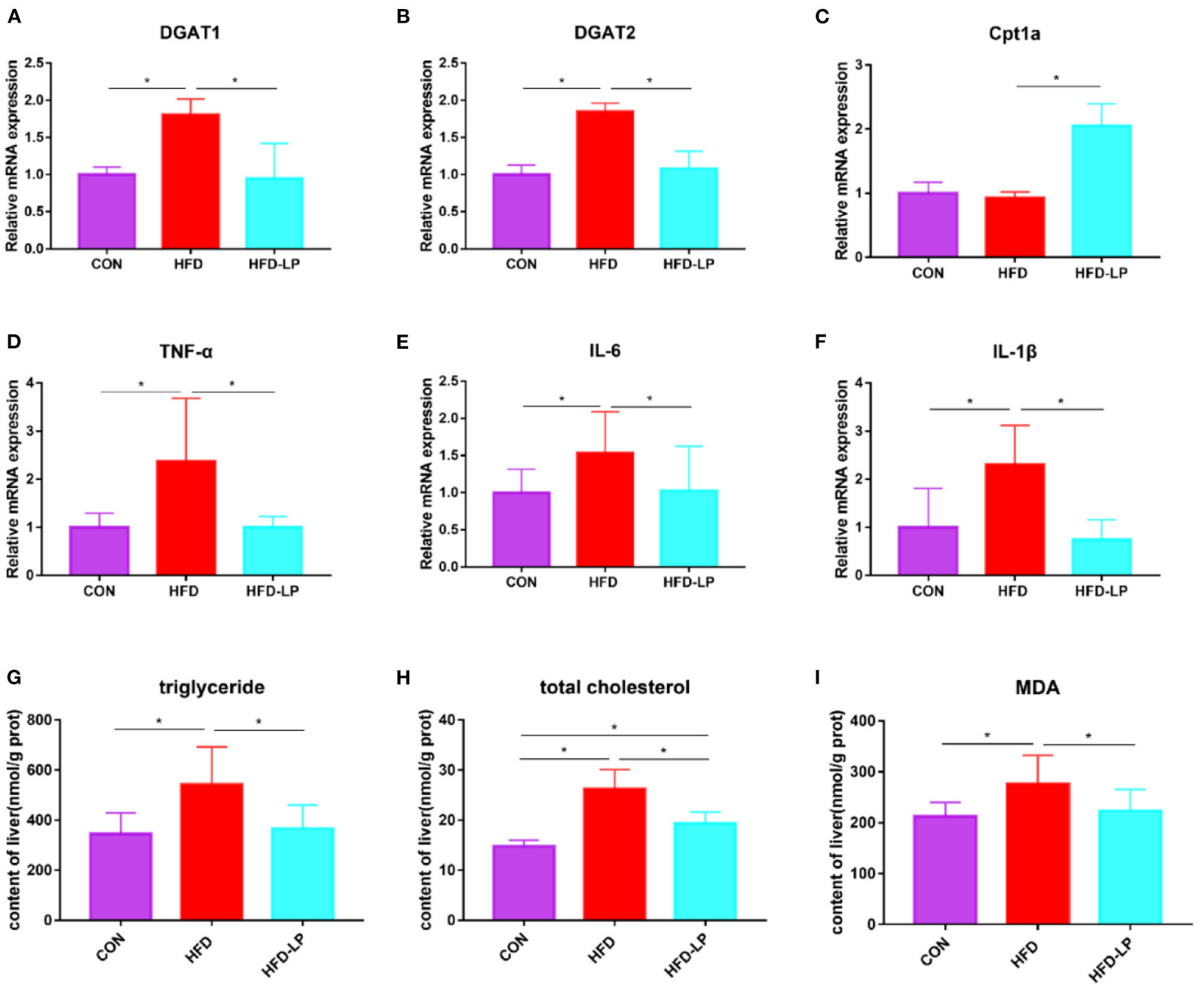
Effects of *Lactobacillus plantarum* on inflammatory factors and lipid metabolism-related genes in obese mice **(A)** relative mRNA expression of DGAT1 in colon tissue; **(B)** relative mRNA expression of DGAT2 in colon tissue; **(C)** relative mRNA expression of Cpt1a in colon tissue; **(D)** relative mRNA expression of TNF-α in colon tissue; **(E)** relative mRNA expression of IL-6 in colon tissue; **(F)** relative mRNA expression of IL-1β in colon tissue; **(G)** the content of triglyceride in liver tissue; **(H)** the content of total cholesterol in liver tissue; **(I)** the content of MDA in liver tissue. Data are mean ± SD (*n* = 8) and analyzed by one-way ANOVA. *p < 0.05.

In order to explore the alleviating effect of *L. plantarum* on chronic liver damage caused by obesity, we measured the expression of inflammatory factors and fat metabolism genes in liver tissues. The results show that compared with the CON and HFD-LP groups, DGAT1, DGAT2, TNF-α, IL-6, and IL-1β mRNA expression in the liver of the HFD group increased significantly (Figures 4A,B,D–F). The expression of Cpt1a in the liver of the HFD-LP group was significantly higher than that in the HFD group (Figure 4C). We also further explored the effect of *L. plantarum* on the ability of the liver to resist oxidative stress. The results showed that high-fat diets significantly increased the levels of triglyceride, total cholesterol, and MDA in the liver tissues of mice. The intragastric treatment of *L. plantarum* significantly reduced the content of these substances and improved the ability of mice to resist oxidative stress (Figures 4G–I).

### *Lactobacillus plantarum* Can Improve SCFAs Content in Mouse Feces

**Figure 5:**
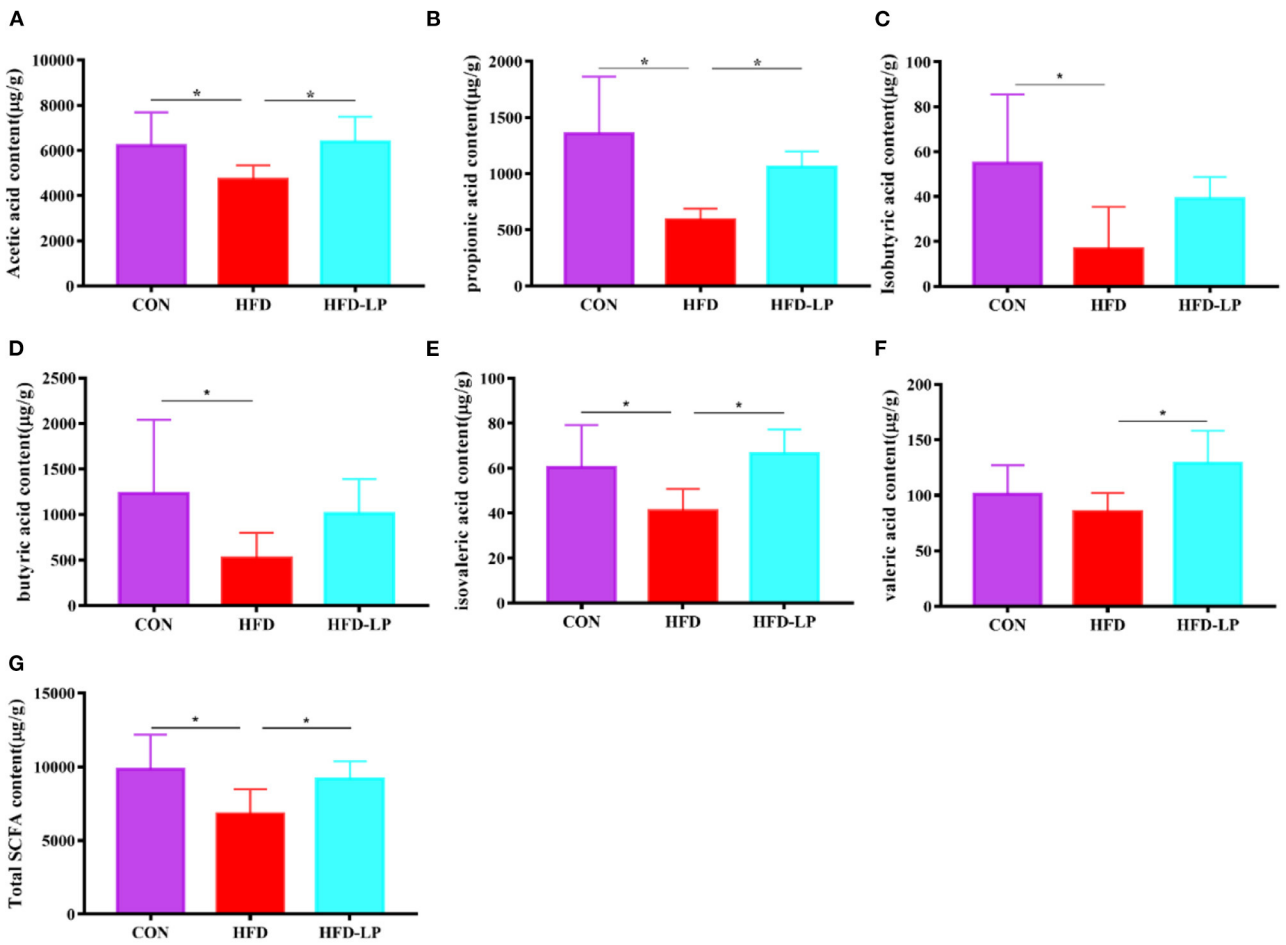
Effect of *Lactobacillus plantarum* on SCFA in obese mice **(A)** the content of acetic acid; **(B)** the content of propionic acid; **(C)** the content of isobutyric acid; **(D)** the content of butyric acid; **(E)** the content of isovaleric acid; **(F)** the content of valeric acid; **(G)** the content of total SCFA. Data are mean ± SD (*n* = 8) and analyzed by one-way ANOVA. *p < 0.05.

As we all know, short-chain fatty acids can regulate the body's metabolism. Therefore, we determined the SCFAs content in feces and explored the regulation effect of *L. plantarum* on host intestinal metabolism. Compared with the CON group, the contents of acetic acid, propionic acid, isobutyric acid, butyric acid, valeric acid and total SCFA in the feces of the HFD group were significantly reduced (Figures 5A–E,G). But compared with the HFD group, the levels of acetic acid, propionic acid, valeric acid, isovaleric acid and total SCFA in the feces of the HFD-LP group increased significantly (Figures 5A,B,E–G).

### *Lactobacillus plantarum* Can Change the Colonic Microbial Composition of Obese Mice

**Figure 6:**
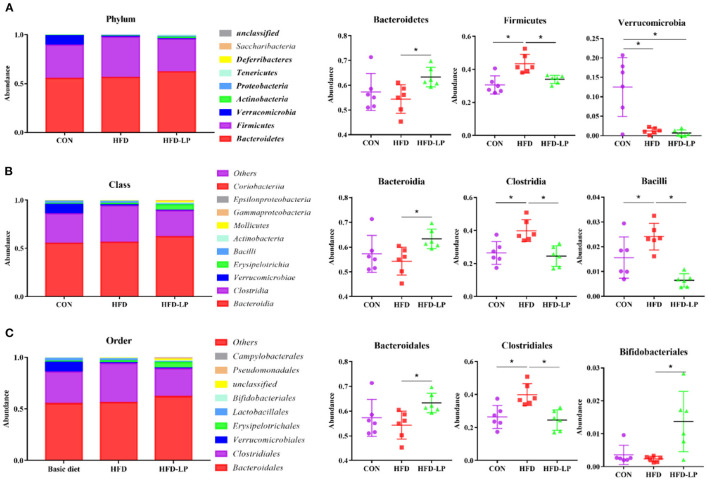
Effect of *Lactobacillus plantarum* on the gut microflora in obese mice **(A)** Microorganism composition structure at phylum level and the specific percentages of *Bacteroidetes, Firmicutes*, and *Verrucomicrobia*; **(B)** Microorganism composition structure at Class level and the specific percentages of *Bacteroidia, Clostridia*, and *Bacilli*; **(C)** Microorganism composition structure at Order level and the specific percentages of *Bacteroidales, Clostridiales*, and *Bifidobacteriales*. Data are mean ± SD (*n* = 6) and analyzed by one-way ANOVA. *p < 0.05.

Finally, we determined the structure of the colonic microbial community to explore the effect of *L. plantarum* on the homeostasis of the mouse intestinal microenvironment. The major bacteria in the colon are *Bacteroidetes, Firmicutes* and *Verrucomicrobia* in the Phylum, accounting for over 97%. The proportion of *Bacteroidetes* is 57.25, 54.39, and 63.28%, that of *Firmicutes* is 30.56, 43.37, and 33.97%, that of *Verrucomicrobia* is 12.50, 1.21, and 0.66% in the CON group, HFD group and HFD-LP group, respectively (Figure 6A). Compared with the CON group, the expression abundance of *Firmicutes* in the colon of the HFD group increased significantly, while the expression abundance of *Verrucomicrobia* decreased significantly (*p* < 0.05). Compared with the HFD group, the expression abundance of *Bacteroidetes* in the colon of the HFD-LP group increased significantly, while the expression abundance of *Firmicutes* decreased significantly (*p* < 0.05). The expression abundance of *Verrucomicrobia* in the colon of the HFD-LP group was significantly lower than that of the CON group (*p* < 0.05).

The top ten bacteria at Class were showed in Figure 6B. The most colonic bacterial are *Bacteroidia, Clostridia* and *Verrucomicrobiae*, accounting for more than 90%. The proportion of *Bacteroidia* is 57.25, 54.29, and 63.27%, that of *Clostridia* is 26.45, 39.92, and 29.49%, that of *Verrucomicrobiae* is 9.37, 0.91, and 0.49% in the CON group, HFD group and HFD-LP group, respectively (Figure 6B). Compared with the CON group, the expression abundance of *Clostridia* and *Bacillli* in the colon of the HFD group increased significantly (*p* < 0.05). Compared with the HFD group, the expression abundance of *Bacteroidetes* in the colon of the HFD-LP group increased significantly, while the expression abundance of *Clostridia* and *Bacillli* decreased significantly (*p* < 0.05).

The top ten bacterial at the Order were selected and made into bar percentages for analysis. The first three were *Bacteroidales, Clostridiales*, and *Verrucomicrobiales*. The proportion of *Bacteroidales* is 57.25, 54.29, and 63.27%, that of *Clostridiales* is 26.45, 39.92, and 24.49%, that of *Verrucomicrobiales* is 9.37, 0.91, and 0.49% in the CON group, HFD group and HFD-LP group, respectively (Figure 6C). Compared with the CON group, the expression abundance of *Clostridiales* in the colon of the HFD group increased significantly (*p* < 0.05). Compared with the HFD group, the expression abundance of *Bacteroidales* and *Bifidobacteriales* in the colon of the HFD-LP group increased significantly, while the expression abundance of *Clostridiales* decreased significantly (*p* < 0.05).

**Figure 7:**
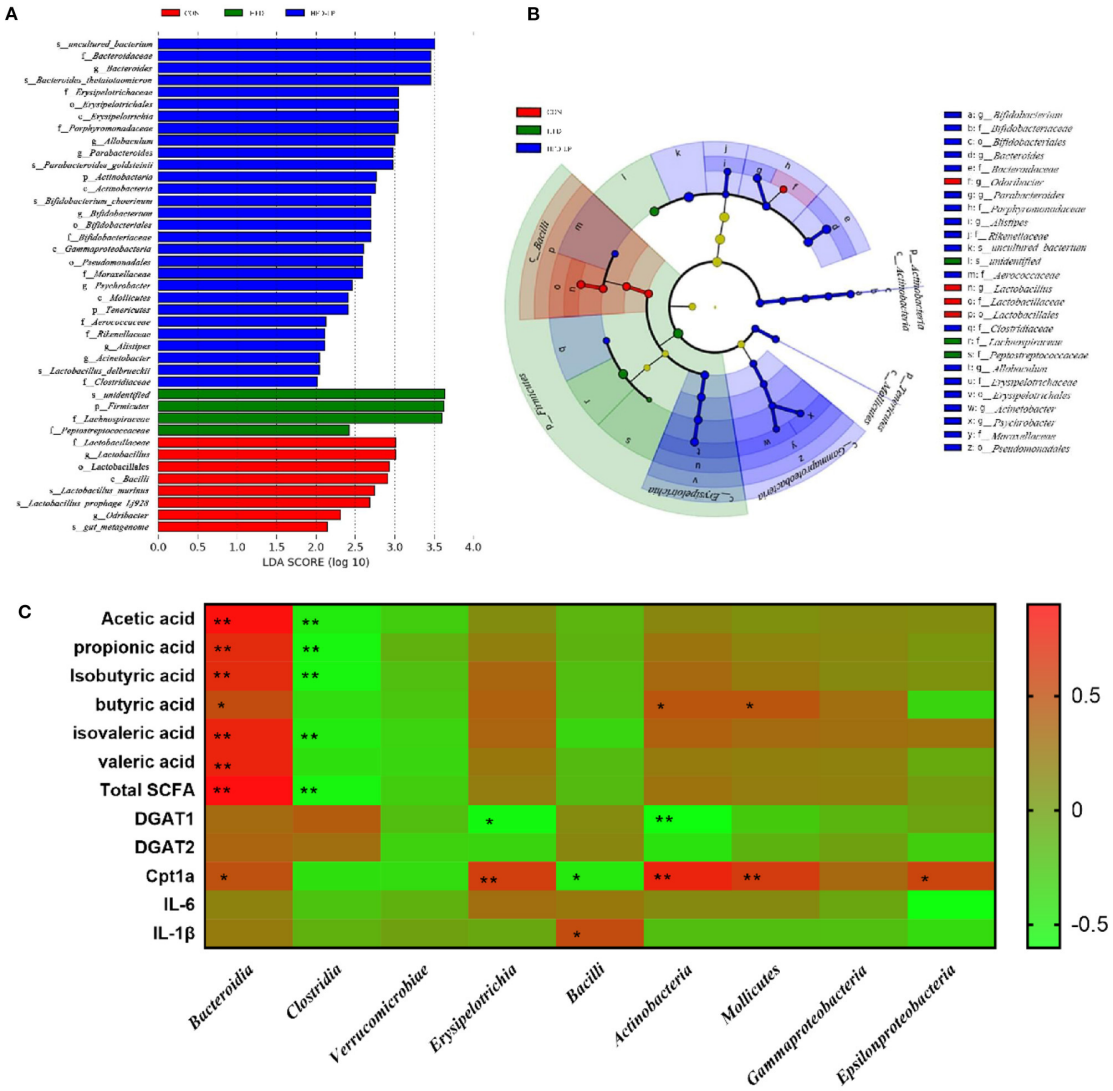
Characteristic of mouse colon microorganisms determined by intragastric administration of *Lactobacillus plantarum*. **(A)** LDA score, an LDA score higher than 2 was considered to be an important contributor to the model; **(B)** LEfSe taxonomic cladogram, different colors suggest enrichment of certain taxa in the control group (red), HFD group (blue) and HFD-LP group (green). **(C)** Analysis of correlations between SCFAs levels, lipid metabolism-related genus level and intestinal microbe abundance.

Through LEfSe analysis, we found that *Bifidobacterium* at the Genus level, *Bifidobacteriaceae* at Family level, and *Bacillales* at Order level were significantly enriched in the HFD-LP group, and had significant effects on differences between groups (Figure 7B, *P* ≤ 0.05). LEfSe then used linear discriminant analysis (LDA) to estimate the magnitude of the effect of species abundance on the effect of differences in each group. We have screened for differential species with LDA scores > 2 as shown in Figure 7A. Finally, we analyzed the correlation between gut microbes and SCFAs, fat metabolism genes and found *Bacteroidia* is positively correlated with all short-chain fatty acids, while *Clostridia* is negatively correlated with five SCFAs, including acetic acid, propionic acid, and isobutyric acid. *Bacteroidia* also showed a positive correlation with Cpt1a (Figure 7C).

## Discussion

Obesity and the chronic metabolic diseases caused by it pose a threat to human health on a large scale in the world (15). We need to find a safe and effective way of treatment and relief, and probiotics are a very effective choice. This is because probiotics have a vital role in gastrointestinal health and the regulation of metabolism (16). In this experiment, we selected a strain of *L. plantarum* with excellent activity and function to explore its effect on lipid metabolism in obese mice. We found that *L. plantarum* can significantly reduce the body weight, fat content, and fat cell size of obese mice. In addition, *L. plantarum* also improves mice's glucose tolerance, insulin resistance, and anti-inflammatory and antioxidant capacity. The most important thing is that it increases the expression of lipid metabolism genes and regulates the morphology of the intestinal microbial community.

Blood glucose is involved in many regulatory processes in lipid metabolism. For example, when glucose concentration in the blood increases, it activates lipid synthetic enzymes, including fatty acid synthase and acetyl-CoA carboxylase, ultimately leading to glucose storage in lipid form in adipocytes (17). In our experiments, obese mice treated with *L. plantarum* gavage showed a significant decrease in blood glucose levels 30 min after receiving intraperitoneal glucose injections, exhibiting the same ability to regulate blood glucose in normal mice. It indicates that obese mice treated with *L. plantarum* gavage can effectively reduce the abnormal increase in blood glucose values and the accumulation of group glucose in the form of fat. On the other hand, insulin can prevent the liver from producing and releasing glucose through glycogenolysis and inhibition of gluconeogenesis. In our experiment, obese mice did not show the hypoglycemic ability of insulin, but mice treated with *L. plantarum* gavage recovered the hypoglycemic function of insulin (18).

Our study is the first to demonstrate that *L. plantarum* can reduce DGAT gene expression in the intestinal tissues of obese mice. The N-terminal region of DGAT1 and the hydrophobic layer form a dimer. The acyl acceptor lipid substrate like DAG enters the lipid bilayer from the water transport channel. The acyl chain of acyl *CoA* is transferred to the receptor, ultimately producing TG (19). Pharmacological manipulations have revealed that DGAT1 activity acts mainly on the gastrointestinal system. The deficiency of DGAT1 in organisms promotes intestinal insulin release and alters lipid absorption, thus improving obesity and its adverse effects (20). And DGAT2 can act synergistically with DGAT1 on TG secretion in hepatocytes (21). Abnormal and excessive accumulation of TGs in organisms can lead to obesity and increase the risk of metabolism-related diseases (22). In our experiments, gavage treatment with *L. plantarum* significantly reduced the abnormal expression of DGAT1 and DGAT2 genes in the intestinal tissues of obese mice and reduced the lipid metabolism-related diseases caused by excessive TG accumulation. Notably, it has been shown that downregulation of the DGAT gene significantly promotes the expression of the CPT1A protein (23), a critical mitochondrial β-oxidation regulatory enzyme involved in long-chain fatty acids, which can dramatically inhibit fat accumulation (24). CPT1A in the outer mitochondrial membrane can catalyze the reverse transport of acyl-coenzyme A groups to L-carnitine to form acyl-carnitine esters (25). In our experiments, gavage treatment with *L. plantarum* significantly increased CPT1A gene expression, promoted fatty acid oxidation, and reduced fat accumulation.

Previous studies have emphasized the important role of SCFA in microbial regulation of intestinal health (26, 27). SCFA is produced by intestinal *Bacteroides* fermenting polysaccharides and some carbohydrates that are difficult for the host to absorb and digest (28). The percentage abundance of *Bacteroides* in the intestine is also increased by the gavage of *L. plantarum*, and as reported in previous experiments, with the increase of the expression level of *Bacteroides*, the gavage treatment of *L. plantarum* also increased the content of SCFA in the content. Acetic acid can promote the oxidation of fatty acids, increase the energy consumption of the host, and ultimately reduce fat (29). The study of Barbara et al. showed that acetic acid activates the 5′-AMP-activated protein kinase in the intestine, thereby mediating adipokines to regulate lipid metabolism and maintain glucose homeostasis (30). On the other hand, SCFA can also enhance the organism's sense of fullness, because acetic acid promotes the circulating levels of GLP-1 and PYY in the body (31).

We have observed that *L. plantarum* can significantly regulate the intestinal microbial community structure of obese mice. It is well known that obese mice fed by HFD have decreased intestinal *Bacteroides* abundance and *Firmicutes* abundance increased (32, 33). However, the ratio of *Firmicutes* to *Bacteroides* returned to normal levels after intragastric treatment with *L. plantarum*. In addition, we found that the gavage treatment of *L. plantarum* also reduced the expression of *Clostridia* in the HFD group. The high abundance of *Clostridia* can metabolize and produce excess fecal deoxycholic acid (DCA) (34). This ecological disorder further reduces the uncoupling of bile acids (35). DCA can stimulate the expression of S1PR2, regulate the protein kinase (ERK) signali pathway, and induce the activation of NLRP3 inflammasomes (36), which is one of the causes of chronic inflammation caused by obesity. This also verified that the gavage treatment of *L. plantarum* can significantly reduce the expression of pro-inflammatory factors in colon tissue. Surprisingly, the gavage of *L. plantarum* significantly improved the expression of *Bifidobacteriales*, and previous studies also showed that it can alleviate obesity (37, 38). *Bifidobacterium* strains can bind to mucin, stimulate the production of colonic mucus, and interact with the intestinal probiotic flora (39). The increased abundance of *bifidobacteria* also prevents the physiological damage of colon mucus and enhances the host body's metabolism (40).

## Data Availability Statement

The datasets presented in this study can be found in online repositories. The names of the repository/repositories and accession number(s) can be found below: https://www.ncbi.nlm.nih.gov/, PRJNA832208.

## Ethics Statement

The animal study was reviewed and approved by the Hunan Agricultural University's Animal Ethics Committee.

## Author Contributions

GL and JF guided and completed the whole experimental design and reviewed and revised the manuscript before submission. YF was involved in the data collection. XH was responsible for the arrangement of data and analyzing the data. YM participated in interpreting the results and wrote the initial draft with all authors providing critical feedback and edits to subsequent revisions. All authors contributed to the article and approved the submitted version.

## Funding

This study was supported by National Natural Science Foundation of China (Nos. 31772642 and 31672457), Ministry of Agricultural of the People's Republic of China (2015-Z64, 2016-X47), Hunan Provincial Science and Technology Department (2021JJ30008, 2019TP2004, 2017NK2322, 2016WK2008, and 2016TP2005), Double First-class Construction project of Hunan Agricultural University (SYL201802003), China Postdoctoral Science Foundation (2018M632963 and 2019T120705), Postgraduate Scientific Research Innovation Project of Hunan Province (CX20210654), and Science and Technology Innovation and Entrepreneurship Project for University Students of Hunan Province (2021RC1004).

## Conflict of Interest

The authors declare that the research was conducted in the absence of any commercial or financial relationships that could be construed as a potential conflict of interest.

## Publisher's Note

All claims expressed in this article are solely those of the authors and do not necessarily represent those of their affiliated organizations, or those of the publisher, the editors and the reviewers. Any product that may be evaluated in this article, or claim that may be made by its manufacturer, is not guaranteed or endorsed by the publisher.
